# The Potential of Curcumin in Treatment of Spinal Cord Injury

**DOI:** 10.1155/2016/9468193

**Published:** 2016-05-19

**Authors:** Raghavendra Sanivarapu, Vijayalakshmi Vallabhaneni, Vivek Verma

**Affiliations:** ^1^Nassau University Medical Center, East Meadow, NY 11554, USA; ^2^Department of Internal Medicine, University of Nebraska Medical Center, Omaha, NE 68198, USA; ^3^Department of Radiation Oncology, University of Nebraska Medical Center, Omaha, NE 68198, USA

## Abstract

Current treatment for spinal cord injury (SCI) is supportive at best; despite great efforts, the lack of better treatment solutions looms large on neurological science and medicine. Curcumin, the active ingredient in turmeric, a spice known for its medicinal and anti-inflammatory properties, has been validated to harbor immense effects for a multitude of inflammatory-based diseases. However, to date there has not been a review on curcumin's effects on SCI. Herein, we systematically review all known data on this topic and juxtapose results of curcumin with standard therapies such as corticosteroids. Because all studies that compare the two show superior results for curcumin over corticosteroids, it could be true that curcumin better acts at the inflammatory source of SCI-mediated neurological injury, although this question remains unanswered in patients. Because curcumin has shown improvements from current standards of care in other diseases with few true treatment options (e.g., osteoarthritis), there is immense potential for this compound in treating SCI. We critically and systematically summarize available data, discuss clinical implications, and propose further testing of this well-tolerated compound in both the preclinical and the clinical realms. Analyzing preclinical data from a clinical perspective, we hope to create awareness of the incredible potential that curcumin shows for SCI in a patient population that direly needs improvements on current therapy.

## 1. Introduction

Spinal cord injury (SCI) is a form of neurological trauma that can be devastating for patients, in part owing to the high rates of disability and resulting medical costs. The most common etiologies of SCI involve trauma; hence, in addition to the 12,400 that are affected yearly in the United States, many more are affected in developing countries as well [1, 2].

Clinical presentation of SCI manifests with a wide variety of neurological deficits, depending on the type and location of injury to the spinal cord (SC). Whereas anteriorly located injuries (anterior cord syndrome) more commonly result in motor deficits, posterior SCI (posterior cord syndrome) results in greater sensory deficits. A third pathology, named central cord syndrome, results in greater motor weakness in upper extremities than in lower extremities, due to anatomic localization of cervical and thoracic tracts closer to the center of the spinal cord.

These clinical symptoms are often the result of profound cellular and molecular alterations in the injured microenvironment. Acute inflammation as well as more delayed glial fibrosis predominates in the injured area, and these factors have made regeneration and therapy after SCI infamously difficult [3–5]. Two phases of SCI exist; primary injury starts after physical impact causing damage to some axons, and the cascade of inflammatory events that follows causes the loss of large numbers of axons resulting in sensorimotor losses, termed secondary injury [6]. Primary physical injury to the SC disrupts cell membranes, destroys myelin and axons, and damages vessels, which in turn triggers inflammatory events and secondary injury. In secondary injury, neutrophils appear at the site of injury within 4 to 6 hours and secrete oxidative and proteolytic enzymes, causing tissue damage [7]. Microglial cells, derived from tissue monocytes, differentiate into macrophages and migrate to the site of injury within 2 days and peak by 5 to 7 days. They further release proinflammatory molecules as well as tissue-damaging reactive oxygen species [8]. These events cause astrocytes to activate and proliferate with overexpression of the glial fibrillary acid protein (GFAP), which contributes to subsequent glial scar formation which is another major deterrent to neuroregeneration.

Current clinical management of SCI is largely supportive. The immediate posttraumatic period can be fraught with cardiovascular and respiratory dysregulation [9, 10]. However, aside from clinical trials showing benefit to corticosteroids as an acute intervention [11–14], and spine surgery if warranted, active interventions are indeed quite few as compared to passive supportive interventions.

Therefore, there is a clear necessity not only for other therapeutic interventions for SCI but also for other modalities of intervention altogether. Natural forms of therapy with minimal to no side effects, as either primary or adjunctive medication for this devastating condition, would be a welcome addition in the clinical arsenal if proven efficacious in preclinical testing. The yellow extract from a rhizome is named* Curcuma longa*; the compound curcumin is a polyphenol substance that has been widely used for medicinal purposes, religious rituals, and local cuisines in the Indian subcontinent. Curcumin has a multitude of effects in the cardiovascular [15], gastrointestinal [16], renal [17], endocrine [18], musculoskeletal [19], and neurologic [20] systems. A central element to curcumin's action on these multiple organ systems is potent inhibition of acute and chronic inflammation [21]. Indeed, the pathogenesis of many chronic diseases is rooted in inflammation. Because inflammation is central to the natural history of SCI, there is deservedly great interest in the potential of curcumin to alleviate the detrimental effects of inflammation and subsequent neurological damage in SCI. Though there are currently no clinical studies, it is a certain hope that the positive results brought forth by this compound in laboratory and translational testing could be eventually used in patients, either on or off a clinical protocol. Because there is currently no summative review in existence that evaluates all available data of curcumin for SCI, we provide the first known systematic review to date in efforts to determine curcumin's role in future clinical testing.

## 2. Materials and Methods

**Figure 1:**
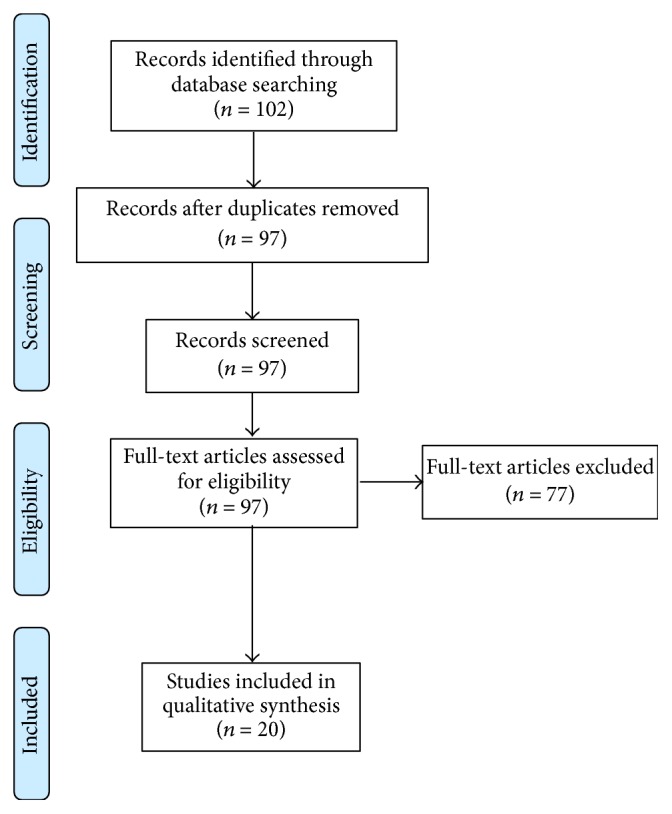
PRISMA diagram illustrating systematic searches for this review.

Eligibility criteria for this systematic review included published work in English evaluating the efficacy of curcumin (turmeric) after spinal cord injury. Sources of information included PubMed, EMBASE, those found in references from the major articles identified, and articles known to the authors. Searches were conducted to identify all articles addressing curcumin for spinal cord injury with the following headings: curcumin, turmeric, diferuloylmethane, spinal cord, and spinal cord injury. Search terms were not restricted in time; all searches were completed by August 1, 2015. Based on initial searches, 102 articles/abstracts were identified. Care was taken to ensure that inclusion criteria were sufficiently broad such that possibly pertinent publications were excluded by individual screening rather than the initial database search. After duplicates were removed, each of the 97 remaining eligible items was independently screened for the criteria described above, and 77 were further excluded. Specifically, articles without specific assessments/reflections on the efficacy of curcumin intervention on SCI, thus being outside the scope of this review, were excluded. Additionally, editorials/commentaries were excluded. Thus, twenty original investigations were found to have sufficient focus and relevance to be incorporated (Figure 1).

## 3. Mobilization of Stem Cells

The spinal cord was not thought to harbor endogenous stem cells until relatively recently [22]. After SCI, it is hence important from a therapeutic perspective to mobilize these stem cells. To this extent, curcumin has previously been found to induce neural stem cell proliferation using stem cells in the brain [23]. However, a group has recently discovered that curcumin stimulates proliferation of neural progenitor cells specifically of the spinal cord [24]. In this work, an extract of spinal cord cells was obtained from experimental rats and was cultured to form neurospheres. The dissociated neuroprogenitor cells were cultured in medium containing different concentrations of curcumin and blank medium as the control. The cell proliferation was quantified at different time periods, and after 48 hours there was enhanced cell proliferation in the low-dose curcumin group as compared to control group. Interestingly, cultures with high-dose curcumin showed decreased proliferation, indicating a dose-dependent action of curcumin in cell proliferation. The authors endorsed that a possible mechanism involves the mitogen activated protein kinase pathway.

Table 1 summarizes these studies. Implications of these findings, if corroborated, are several. First, if curcumin can be utilized to induce neural stem cell proliferation of the SC in vivo, there may be less of a necessity to externally implant stem cells in SCI models, which though efficacious, would need greater levels and time of clinical testing. Second, the endogenous progenitors that do proliferate in the SC after SCI [25] may be augmented by curcumin, which would be an important finding for future research to document. Lastly, it will also be essential to assess whether curcumin causes differential proliferation in neural progenitors as opposed to largely unwanted glial cells, which are known to cause barriers in post-SCI recovery [3–5].

## 4. Antioxidant Effects

SCI has been well-associated with increased free radical production, likely as a result of inflammation [7, 8]. Free radicals have unpaired electrons in their valence orbit and thus look to react with nearby tissue, which causes cytoarchitectural damage. Curcumin has been shown in multiple studies to offer antioxidant properties (Table 2). In one report [26], SCI rats were subjected to decompressive laminectomy alone, surgery and curcumin, or surgery with methylprednisolone (a corticosteroid). Blood levels of the antioxidant superoxide dismutase (SOD) and the oxidative agent malondialdehyde (MDA) were measured after 24 hours. The results showed higher SOD levels and lower MDA levels in the curcumin group as compared to both the control and methylprednisolone groups. These results are particularly interesting in light of the fact that methylprednisolone is very often used in clinical SCI treatment. It suggests that the corticosteroid may be a symptomatic measure but not necessarily address the cellular and molecular aspects of SCI-mediated injury.

A short paper evaluated one known effect of free radicals, lipid peroxidation [27]. In also examining rats treated with curcumin and methylprednisolone, though the results did not point to improved neurological recovery with curcumin over methylprednisolone (a finding contrary to all available publications), the authors did observe that lipid peroxidation is decreased in the curcumin-treated rats. Hence, intuitively, one would not expect that corticosteroids decrease free radicals, and this remains an advantage of curcumin over existing therapies.

Another report examined antioxidant effects of curcumin specifically in the reperfusion phase of ischemic SCI [28]. Though ischemia is a relatively uncommon etiology of SCI, inflammation-induced neurological damage remains a central aspect of pathogenesis in both traumatic and nontraumatic SCI. Rabbits were subjected to transient spinal cord ischemia and a subpopulation was given curcumin. The results a mere 48 hours after treatment with curcumin showed statistically significant improvement in neurological function, reduced apoptosis, and lowered MDA levels with increased SOD activity. Hence, this report illustrated the consistency of curcumin to produce antioxidation in both traumatic and nontraumatic models of SCI.

A systematic review and network meta-analysis has also addressed curcumin's antioxidant effects [29]. Not only does curcumin decrease MDA levels, it also induces neurological recovery in a dose-dependent manner, utilizing a locomotor scoring method. Though the concept of curcumin and neurologic improvements will be discussed in a subsequent section, these results are significant for elucidating that though causation cannot be implied, the “clinical rewards” of tipping the oxidation-antioxidation balance towards the latter could be associated with enhanced functional recovery.

## 5. Decreasing Inflammation and Fibrosis

The major mechanism impeding neurological recovery after SCI is the uncontrolled inflammation and glial-mediated scarring, which creates an antineurogenic niche that is vastly better described in experiments of the brain as compared with SC [30]. Occurring concurrently with acute inflammation and preceding fibrosis, spinal cord edema plays a large role in neurologic damage and patient symptoms, which is a main reason why corticosteroids are clinically used in the management of SCI patients. Though not without methodological flaws, a study examined tissue edema in rats with chronic constrictive SCI models treated with or without curcumin [31]. The edematous content in damaged SCs was reduced significantly in curcumin-treated rats, along with decreased expression of the water-obtaining protein aquaporin. Associated with decreased tissue edema was the finding of improved neuromotor activity in the curcumin-treated rats.

Results to support this finding exist from other data that examined both inflammation and fibrosis in mice with laminectomy-induced SCI [32]. Mice were given either adjuvant curcumin, dimethyl sulfoxide (control), or sham surgery only. In the curcumin group, there were several important findings of note. First, the curcumin-treated mice had decreased expression of NF-*κ*B (a proinflammatory cytokine known to be inhibited by curcumin) and Iba-1 (a marker for inflammatory microglia). Second, GFAP expression and glial scars were also reduced in curcumin-treated mice. Lastly, neurofilament-200 expression (a marker for neurons) was dramatically increased in the curcumin group, suggesting that more native neurons remained in mice treated with curcumin, although it is illogical to conclude that the simple presence of neurons in postinjury SCs could be similarly functional as preinjury circumstances.

Corroborative results were found in another report that demonstrated neuroprotective effects of curcumin [33]. Herein, rats with experimentally induced hemisectioned SCs were randomized into sham, vehicle, and curcumin groups. The recovery of motor function and glial activity after SCI were examined, which showed decreased GFAP expression as detected by polymerase chain reaction. Similar to the previous data, decreased neuronal loss in the curcumin group was also evidenced. Functional outcomes by the standard scaling systems as aforementioned studies also supported their results of enhanced recovery with curcumin.

An intriguing recent study [34] is the first to assess inflammatory and fibrotic parameters in rats treated with curcumin versus methylprednisolone. The spinal cord cavities were compared among all groups (including vehicle and sham groups) for expression of the inflammatory and fibrosis-related molecules TNF-*α*, IL-1*β*, NF-*κ*B, GFAP, TGF-*β*1, TGF-*β*2, and SOX-9. In curcumin-treated rats, there was reduced expression of all the aforementioned proteins, along with decreased extracellular matrix deposition (prescarring). These observations had a dose-dependent effect with higher doses leading to more significant effects. Importantly, the methylprednisolone treated groups had levels of the aforementioned parameters that were greater than the control groups but less than the curcumin group. These results, especially if validated, have large implications on potential future clinical practice. Similar to a previously discussed paper [26], if curcumin shows decreased cellular and molecular profiles of inflammation and fibrosis as compared with established clinical pharmaceuticals (e.g., methylprednisolone), then it likely has clinical effects at minimum equivalent to corticosteroids. That curcumin has showed that high-dose tolerance—up to 12,000 mg curcumin per day—certainly beckons phase I-II trial testing [35]. Tabulated preclinical studies examining inflammation and fibrosis are given in Table 3.

## 6. Induction of Functional Neurologic Recovery

Though many previous pertinent results have been already mentioned, others will be presented in greater detail hereafter (Table 4). An investigation from New York Medical College used traumatic SCI rat models receiving curcumin or dimethyl sulfoxide within 30 minutes after contusion and weekly thereafter for 6 weeks via percutaneous epidural injections [36]. SCI recovery was assessed using standard aforementioned scoring systems. Throughout the study, motor scores of the curcumin group were higher than the control group. The group used body weight as a surrogate marker for recovery, and this was also increased in curcumin-treated rats. At 6 weeks, soleus muscle weights were significantly higher as well, and histopathological quantitative analysis revealed greater grey matter sparing in the curcumin group, coinciding with less gliosis. Similar results have been strongly indicative of validation in other studies as well which have used similar, yet different, methodologies, suggesting consistency of curcumin in inducing neuromotor recovery [37]. A strength of many studies examining functional recovery is that a uniform scale (the Basso-Beattie-Bresnahan score) is used for interstudy comparison, making extrapolations at least somewhat more reliable than utilizing different scales.

An ischemic SCI model was established in another publication, testing similar parameters [38]. In this study, after ligating the specific vasculature, rats treated with intraperitoneal curcumin showed downregulated inducible nitric oxide synthase and N-methyl aspartate receptor expression; these serve as reliable surrogate markers of excitotoxicity caused by vasogenic inflammation. Though this paper used a different neuromotor scale than most other papers, results were similarly favorable for curcumin. Therefore, together with traumatic SCI models, nontraumatic models of SCI also result in similar successes for curcumin in animal models.

Though curcumin is known to act in multiple pathways, several of which have been previously discussed, another group has demonstrated that calcitonin gene-related peptide (CGRP) expression is related to improvement in neuromotor function [39]. Rat models of SCI were given low and high doses of curcumin and were compared with sham and control groups. Outcome measures included CGRP expression and motor function scores, both with standard scoring as well as the inclined plane test. Both the curcumin groups showed superior neuromotor scores as well as CGRP-positive cells. Further testing will be needed to ascertain various effector-mediated mechanisms of curcumin in vivo.

A large part of healthcare costs and patient morbidities after SCI relate to persistent and chronic neurogenic pain after the insult, and hence a group evaluated curcumin as antinociceptive therapy in murine SCI models [40, 41]. In fact, the novel curcuminoid KMS4034 was used, owing to its greater bioavailability. The mice were subjected to subcutaneous injections of formalin in order to assess the duration of paw flinches, licking, and biting in curcumin and control groups. Decreased licking and flinching durations indicate greater relief of pain, which were seen after curcuminoid administration, as well as concurrent administration of gabapentin, a medication used clinically for a wide variety of nerve-related pathologies. The hot plate test was done to evaluate acute pain; the withdrawal latency to noxious stimuli in mice treated with the curcuminoid was reduced compared to control animals. Though the paper also delves into mechanisms, neuropathic pain mechanisms are immensely complicated and a thorough discussion is beyond the scope of this review. These authors do note that one potential mechanism of the analgesic effect of curcumin is through modulating TRPV1, a ligand gated calcium ion channel involved in nociceptive signaling as well as the descending monoamine system. Though this necessitates further study, the results of this paper clearly show that nociception can indeed be improved in animals and warrants further testing in humans, either empirically or with trials in hopes for similar benefits. Demonstration of even slight improvement in humans could potentially go a long way in relieving morbidities and healthcare costs in this patient population.

A major emphasis of neurorehabilitation after SCI is for the patient to mobilize and aggressively undergo physical therapy as much as possible, especially early after the event. A group at the University of California, Los Angeles, examined rats with transected SCs and investigated sensorimotor learning in four groups of rats while altering two variables [42]. Diet was varied from control to that containing curcumin and the omega-3 fatty acid DHA and activity levels being either sedentary or with exercise. Improved functional learning outcomes were observed in those rats given DHA and curcumin, irrespective of exercise level. Though molecular mechanisms were proposed, the most important was thought to be brain-derived neurotrophic factor, syntaxin-3, and decreasing lipid peroxidation, which are thought to be involved in physical conditioning and maintaining “muscle learning” in these rats [43]. It is intriguing to examine from a therapeutic perspective that the dogma of aggressive exercise/rehabilitation did not show improvements as much as dietary omega-3 and curcumin (although exercise and rehabilitation in animal models are clearly unequal). Though the role of omega-3 in these patients remains relatively equally less-defined, these data also are notable for involving oral intake of curcumin, from which traditionally scientists have shied away owing to poor bioavailability. Further analyses are needed in order to determine whether oral curcumin—most practical in human subjects—can suffice in lieu of parenteral administration. Additionally, it cannot even be currently determined whether curcumin's main actions are on the SC itself, vasculature, or downstream peripheral nerves from the degenerating SC—in other studies of curcumin in chronic constriction of the sciatic nerve with intact SCs, the compound delivers nearly equal functional recovery as SCI models [44]. Further analyses must be conducted in order to determine these answers.

Lastly, emerging modalities of cell therapy for neurodegenerative conditions have shown great promise for brain regeneration, but regeneration after SCI in conjunction with curcumin has to date been shown in only one report [45]. After isolating neural stem cells from the subventricular zone of the brain, curcumin was shown to induce greater proliferation at low doses only (consistent with other studies [24]). SCI was defined in this paper as moderate or severe depending on the distance of the traumatic weight drop, simulating crush injury on the SC. Whereas after moderate SCI, salubrious effects of neural stem cells and curcumin together were similar to the cells alone, severe SCI showed synergistic effects of curcumin with the stem cells. These synergistic results in severe SCI were also seen in the body weight and soleus muscle weight parameters as well. These data are extremely intriguing not only for regenerative scientists but also for clinicians interested in cell therapy for use in the clinic, which have been accomplished with positive clinical results in the setting of another neurodegenerative disease, Parkinson's [46]. Because cell therapy faces challenges of its own, such as suboptimal postimplantation cell survival and integration, molecules that could aid implanted cells are certainly a welcome notion.

## 7. Future Directions

SCI is a substantial health epidemic throughout the world, and the relative stagnation of developments past corticosteroid therapies necessitates other routes of improving function and quality of life in these patients [47]. There are several methods being utilized as experimental therapies for patients with SCI, including electrical nerve stimulation [48], therapeutic hypothermia [49], and cell therapy [50]. We propose the benefit of adding curcumin to these methods, not only because of high, albeit untested, likelihood for functional improvements, but also because of the fact that curcumin acts at the central pathogenesis of SCI-induced neurological injury—inflammation.

Many questions remain in need of greater assessment before curcumin can transition into the clinical realm. First, the bioavailability of oral curcumin is notoriously low, but despite increasing with lipid intake, would it be enough to make a clinical impact? It has been established that the lipophilic curcumin can penetrate the blood-brain barrier and percolate into the CSF, which is the primary route by which neuroprotection is largely mediated [51, 52]. Studies in osteoarthritis have shown great promise with conjugated delivery forms, having outpaced current standards of care in patients with osteoarthritis [53]. These formulations can also greatly augment CNS biodistribution of curcumin, as expounded by Tsai and colleagues [54]. Second, with high-dose tolerance and essentially no side effects of curcumin, is it feasible to deliver megadoses of curcumin parenterally in patients? There are currently several parenteral formulations of curcumin, including the trademark names known as Lipocurc*™*, NanoCurc*™*, Meriva*™*, and CurcuVET*™* [55–59]. Though these are not approved by the USA Food and Drug Administration, is it also feasible to take megadoses of oral curcumin (with appropriate lipid-laden meals to increase gastrointestinal absorption) after SCI—in a sense of “off-label” and empiric use? In turn, would curcumin and corticosteroids make a greater clinical impact than the latter alone? Third, can the future bring more corroborative evidence of the effects of curcumin when given in a delayed setting? Though only a few studies herein administered curcumin at ≥ 24 hours after SCI, it will be important to more precisely evaluate curcumin's contributions if administered in the nonacute setting. Lastly, can clinicians shed the “herbal medicine” stigma and be able to acknowledge that ignoring its beneficial effects is largely due to a lack of clinical data and not necessarily a result of inferior clinical efficacy?

## Figures and Tables

**Table 1:** Publications examining the stem/progenitor cell proliferative properties of curcumin. BrdU, bromodeoxyuridine; *μ*M, micromolar.

Reference	Cell type	Animal type	Study design	Curcumin administration	Outcomes/results
Hucklenbroich et al. [23]	Fetal rat neural stem cells	Wistar rats	In vitro (in cell cultures) & in vivo (six control, three treated) examination of proliferation (BrdU immunofluorescence)	In culture medium at various concentrations (1–25 *μ*M); intracerebroventricular injection in rats	Curcumin with increased proliferation in vitro at lower doses (1–6 *μ*M), decrease at higher dose levels (12–25 *μ*M) In vivo, curcumin induced increased proliferation, with majority of subsequent differentiation into neurons/neuroblasts

Son et al. [24]	Neural progenitor cells from Sprague-Dawley rat spinal cords	—	Examine cellular proliferation (MTS assay) between control (no curcumin) and curcumin at 6 different dose levels	In culture medium at 0.1, 0.5, 1, 10, 20, and 50 *μ*M	Lower doses (0.1, 0.5, and 1 *μ*M) increase proliferation; higher doses (10, 20, and 50 *μ*M) decrease proliferation; potential mechanism via mitogen-associated protein kinase pathway

**Table 2:** Publications examining the antioxidant properties of curcumin. Groups equally divided unless otherwise indicated. SCI, spinal cord injury; SOD, superoxide dismutase (antioxidant); MDA, malondialdehyde (oxidant); DMSO, dimethyl sulfoxide.

Reference	Sample size	Animal type	Method of SCI	Treatment groups	Route & timing of curcumin administration	Pathological findings	Outcomes/results
Şahin Kavakli et al. [26]	24	Wistar rats	Weight drop	After SCI: curcumin, methylprednisolone, or control	Orally; continuously until sacrificing	—	Higher SOD levels in curcumin group versus other two groups; lower MDA levels in curcumin & methylprednisolone groups versus control

Sanli et al. [27]	40	Wistar rats	Weight drop	No SCI, SCI alone, SCI/DMSO, SCI/curcumin/DMSO, and SCI/methylprednisolone	Single intraperitoneal dose directly after SCI	No difference in caliber of myelinated axons; less mitochondrial trauma in curcumin, methylprednisolone, and DMSO groups	Decreased lipid peroxidation and MDA levels in curcumin group; curcumin, methylprednisolone, and DMSO groups with improved neurological/functional tests

Liu et al. [28]	36	New Zealand rabbits	Transient (30 min) abdominal aortic occlusion	Sham SCI, SCI only, and SCI/curcumin	Single venous injection 10 minutes prior to SCI	Greater histologically normal neurons & fewer apoptotic cells in curcumin group	Improved neurological (motor) function in curcumin group

Yao et al. [29]	8	—	Various	Meta-analysis of 8 studies of curcumin versus control	Various	—	Curcumin-treated animals with lesser MDA levels and improved neuromotor functioning with potential dose response

**Table 3:** Publications examining the anti-inflammatory and fibrotic properties of curcumin. Groups equally divided unless otherwise indicated. SCI, spinal cord injury; DMSO, dimethyl sulfoxide; AQP-4, aquaporin-4; GFAP, glial fibrillary acidic protein (astrocyte marker); pJAK-STAT, phosphorylated Janus kinase-signal transducers and activators of transcription; Iba-1 (microglial marker); NF-200, neurofilament-200 (neuron marker); IL-1*β*, interleukin-1*β*N (inflammatory mediator); NO, nitric oxide (inflammatory mediator); NF-*κ*B, nuclear factor-*κ*B (inflammatory mediator); NeuN, neuronal nuclei (neuron marker).

Reference	Sample size	Animal type	Method of SCI	Treatment groups	Route & timing of curcumin administration	Pathological findings	Outcomes/results
Zu et al. [31]	64	Sprague-Dawley rats	Weight drop	Sham/DMSO, sham/curcumin, SCI/DMSO, and SCI/curcumin	Single intraperitoneal injection 30 minutes after SCI	In curcumin/SCI versus SCI/DMSO group, increased gray-white matter interface, tissue edema/AQP-4 expression, as well as GFAP/pJAK-STAT expression	Functional motor scores higher in SCI/curcumin group than in SCI/DMSO group

Wang et al. [32]	Not specified	BALB/c mice	Extradural clip for 3 seconds	Sham, SCI/DMSO, and SCI/curcumin	Single intraperitoneal injection immediately after SCI	In SCI/curcumin versus SCI/DMSO group, decreased tissue expression of GFAP & Iba-1 and increased NF-200	With curcumin, decreased levels of IL-1*β*, NO, and NF-*κ*B; increased neuromotor scores

Lin et al. [33]	39	Sprague-Dawley rats	Spinal cord hemisection	Sham (*n* = 5), SCI/DMSO (*n* = 17), and SCI/curcumin (*n* = 17)	Daily intraperitoneal injection beginning 1 day after SCI, for 6 total days	In SCI/curcumin versus SCI/DMSO group, fewer apoptotic & GFAP cells and more NeuN/cells	Improvement in motor performance in SCI/curcumin group over SCI/DMSO group at days 3 & 7

Yuan et al. [34]	36	Sprague-Dawley rats	Clip for 60 seconds	Sham, SCI only, SCI/curcumin (three dose levels), and SCI/methylprednisolone	Immediate intraperitoneal injection after SCI, followed by daily injections for 7 total days	Over other groups, smaller histological glial scar and GFAP expression in SCI/curcumin group, with numerical dose response	Decreased expression of several inflammatory and fibrotic cytokines, viable axonal fibers, and functional recovery in SCI/curcumin group over other groups, no appreciable dose response

**Table 4:** Publications examining effects of curcumin on functional neurological recovery after spinal cord injury. Groups equally divided unless otherwise indicated. SCI, spinal cord injury; DMSO, dimethyl sulfoxide; SOD, superoxide dismutase (antioxidant); MDA, malondialdehyde (oxidant); iNOS, inducible nitric oxide synthase; NMDA, N-methyl-D-aspartate; CGRP, calcitonin gene-related peptide; KMS4034, curcumin analog; DHA, docosahexaenoic acid; NSCs, neural stem cells.

Reference	Sample size	Animal type	Method of SCI	Treatment groups	Route & timing of curcumin administration	Pathological findings	Outcomes/results
Ormond et al. [36]	14	Sprague-Dawley rats	Weight drop	After SCI: curcumin versus DMSO	Epidural injection within 30 minutes of SCI & weekly thereafter, until sixth week	Curcumin group with greater spinal cord tissue sparing & neuronal tissue sparing	In curcumin group, improved functional scores after 3 weeks and greater soleus weight

Kim et al. [37]	36	Sprague-Dawley rats	Clipping for 120 seconds	Sham, SCI/vehicle, and SCI/curcumin	Seven consecutive days of intraperitoneal injections after SCI	Curcumin group with decreased cavity size two weeks after SCI	In curcumin group, higher neuromotor scores after 7 days; increased SOD and decreased MDA and macrophage markers

Zhang et al. [38]	30	Sprague-Dawley rats	Permanent ligation of lumbar artery	Sham, SCI/saline, and SCI/curcumin	Intraperitoneal injection daily for 7 days starting 24 hours after SCI	Curcumin group with decreased iNOS and NMDA expression as compared with saline group	Higher hind limb motor function in curcumin group at 7 days

Sun and Xu [39]	200	Rats, unknown type	Not specified	Sham, SCI/saline, SCI/low-dose curcumin, and SCI/high-dose curcumin	Intraperitoneal injection immediately after SCI	Curcumin group with increase in CGRP+ cells, starting at 3 days, with dose response	Enhanced motor scores of curcumin-treated groups starting at 3 days; dose response between high- and low-dose curcumin groups starting at 7 days

Lee et al. [40]	40	ICR mice	Monofilament-based chronic constriction injury	Vehicle, gabapentin (positive control), and KMS4034 (0.1, 1, and 10 mg/kg)	Not specified	10 mg/kg KMS4034 group with decrease in CGRP+ cells	KMS4034 and gabapentin groups with decreased postnoxious stimulus paw licking, flinching, and withdrawal latency

Zhao et al. [41]	Not specified	C57BL/6J mice	Chronic constrictive injury of sciatic nerve	Vehicle, curcumin at various dose levels (5, 15, and 45 mg/kg)	Orally, twice daily for three weeks starting 10 days after SCI	—	Decreased mechanical allodynia and thermal hyperalgesia in curcumin groups with dose response; effects abrogated with impedance of monoamine signaling

Joseph et al. [42]	52	C57BL/6J mice	Spinal cord transection	Control diet/sedentary, control diet/exercise, DHA/curcumin/sedentary, and DHA/curcumin/exercise	21 days prior to intervention; diet ad libitum	—	Enhanced spinal cord motor learning in both curcumin/DHA groups and highest in group with exercise; effects mediated by several signaling factors including neurotrophic factors

Holly et al. [43]	27	Sprague-Dawley rats	Placement of paraspinal nonresorbable polymer	Control (no SCI), SCI/Western diet, and SCI/DHA/curcumin	Diet ad libitum and for 6 weeks after procedure	—	Improved gait at 3 & 6 weeks in the DHA/curcumin groups; potential mediation via neurotrophic factors

Di et al. [44]	72	Sprague-Dawley rats	Chronic constrictive injury of sciatic nerve	Sham, SCI only, SCI/vehicle, and SCI/curcumin	Intraperitoneal injection daily for 14 days, starting 1 day after SCI	Decreased corticosteroid synthesis/expression in curcumin group	Curcumin group with improved withdrawal to thermal stimulation, starting at 7 days

Ormond et al. [45]	63	Sprague-Dawley rats	Weight drop from close (moderate SCI) or afar (severe SCI)	SCI only (*n* = 18), SCI/curcumin (*n* = 10), SCI/NSCs (*n* = 16), and SCI/curcumin/NSCs (*n* = 19)	Intramuscular injection near site of injury, within 20 minutes	Numerically greatest spinal cord parenchymal sparing with curcumin/NSCs	Synergistic effect of curcumin and NSCs in neuromotor recovery, along with body weight after SCI and soleus muscle weight (after severe SCI)

## Abbreviations

SCI: Spinal cord injury
SC: Spinal cord
GFAP: Glial fibrillary acidic protein
SOD: Superoxide dismutase
MDA: Malondialdehyde
CGRP: Calcitonin gene-related peptide.

## Additional Points

(i) Spinal cord injury (SCI) is a type of neurotrauma characterized by acute and chronic inflammation and delayed scarring; there are currently no major options for clinical management other than supportive care and perhaps corticosteroids. (ii) Curcumin is a natural compound known to have many anti-inflammatory and anticancer properties, although a dearth of data, especially summative data, exist on its role in ameliorating SCI. (iii) Curcumin has been shown to mobilize neural stem cells of the spinal cord and provide antioxidant effects after SCI's free radical-mediated damage to the spinal cord. (iv) Curcumin's powerful anti-inflammatory activity in the post-SCI environment has been shown to translate to decreased inflammatory cells and proinflammatory molecules. (v) In animal studies, curcumin has substantially improved functional neurologic recovery after SCI; moreover, on both the microscopic and macroscopic levels, curcumin has been shown in multiple publications to be an improvement over corticosteroids alone. (vi) These encouraging laboratory data need to be translated to the clinical realm for further testing, as well as corroboration in the laboratory as well.
